# Comparison of neuromuscular and quadriceps strengthening exercise in the treatment of varus malaligned knees with medial knee osteoarthritis: a randomised controlled trial protocol

**DOI:** 10.1186/1471-2474-12-276

**Published:** 2011-12-05

**Authors:** Kim L Bennell, Thorlene Egerton, Tim V Wrigley, Paul W Hodges, Michael Hunt, Ewa M Roos, Mary Kyriakides, Ben Metcalf, Andrew Forbes, Eva Ageberg, Rana S Hinman

**Affiliations:** 1 The University of Melbourne, Centre for Health, Exercise and Sports Medicine, Department of Physiotherapy, School of Health Sciences, Melbourne, Vic, Australia; 2 The University of Queensland, School of Health and Rehabilitation Sciences, St Lucia, Brisbane, QLD, Australia; 3 University of British Columbia, Department of Physical Therapy, Vancouver, BC, Canada; 4 University of Southern Denmark, Institute of Sports Science and Clinical Biomechanics, Odense, Denmark; 5 Monash University, Department of Epidemiology and Preventive Medicine, Melbourne, Vic, Australia; 6 Lund University, Department of Orthopedics, Clinical Sciences Lund, Lund, Sweden; 7 Lund University, Department of Health Sciences, Lund, Sweden

## Abstract

**Background:**

Osteoarthritis of the knee involving predominantly the medial tibiofemoral compartment is common in older people, giving rise to pain and loss of function. Many people experience progressive worsening of the disease over time, particularly those with varus malalignment and increased medial knee joint load. Therefore, interventions that can reduce excessive medial knee loading may be beneficial in reducing the risk of structural progression. Traditional quadriceps strengthening can improve pain and function in people with knee osteoarthritis but does not appear to reduce medial knee load. A neuromuscular exercise program, emphasising optimal alignment of the trunk and lower limb joints relative to one another, as well as quality of movement performance, while dynamically and functionally strengthening the lower limb muscles, may be able to reduce medial knee load. Such a program may also be superior to traditional quadriceps strengthening with respect to improved pain and physical function because of the functional and dynamic nature. This randomised controlled trial will investigate the effect of a neuromuscular exercise program on medial knee joint loading, pain and function in individuals with medial knee joint osteoarthritis. We hypothesise that the neuromuscular program will reduce medial knee load as well as pain and functional limitations to a greater extent than a traditional quadriceps strengthening program.

**Methods/Design:**

100 people with medial knee pain, radiographic medial compartment osteoarthritis and varus malalignment will be recruited and randomly allocated to one of two 12-week exercise programs: quadriceps strengthening or neuromuscular exercise. Each program will involve 14 supervised exercise sessions with a physiotherapist plus four unsupervised sessions per week at home. The primary outcomes are medial knee load during walking (the peak external knee adduction moment from 3D gait analysis), pain, and self-reported physical function measured at baseline and immediately following the program. Secondary outcomes include the external knee adduction moment angular impulse, electromyographic muscle activation patterns, knee and hip muscle strength, balance, functional ability, and quality-of-life.

**Discussion:**

The findings will help determine whether neuromuscular exercise is superior to traditional quadriceps strengthening regarding effects on knee load, pain and physical function in people with medial knee osteoarthritis and varus malalignment.

**Trial Registration:**

Australian New Zealand Clinical Trials Registry reference: ACTRN12610000660088

## Background

Knee osteoarthritis (OA) is a common chronic joint disease and costly public health problem. It leads to pain, loss of function and reduced quality-of-life [1]. The economic impact of knee OA is substantial and will further increase as the population ages and obesity rates escalate [2-4]. There is no cure for the condition and typically about one third of people with knee OA will experience structural deterioration [5] with many of these ultimately requiring knee joint replacement surgery [6].

Knee OA usually affects the medial tibiofemoral joint compartment [7], probably because of the increased load borne on this compartment during normal walking [8]. Around three quarters of people with knee OA have varus malalignment measured statically on x-ray [9]. Increasing varus malalignment usually occurs because of progressive loss of cartilage and joint space in this compartment. People with medial knee OA and varus malalignment exhibit unique characteristics and responses to treatment compared to people with more neutral alignment. These individuals show greater functional decline over time [10] and are at a 3- to 4-fold greater risk of structural disease progression than those with more neutrally aligned knees [11]. Importantly, quadriceps strengthening exercise, a cornerstone of traditional treatment for OA, has been shown to be ineffective at reducing pain in people with varus malalignment [12]. Thus, there is a need to develop and evaluate interventions for this particular sub-group of people with knee OA.

The poorer prognosis for people with medial knee OA and varus malalignment is likely due to the greater compressive load borne on the diseased medial compartment in these people compared to those with more neutrally aligned knees. Varus malalignment causes the ground reaction force vector to pass more medially to the knee joint centre during gait resulting in increased loads across the medial compartment [8]. Higher compressive knee loads are implicated in knee OA development and progression. This is highlighted by *in vivo *animal experiments [13], and by the positive relationship of knee OA to obesity [14,15] and occupations involving heavy lifting or prolonged kneeling or squatting [16].

Three dimensional gait analysis is typically used to infer compressive joint loads. The most widely studied parameter in knee OA is the external knee adduction moment (KAM) [17]. The KAM tends to force the knee into varus and thus compresses the medial joint compartment and stretches lateral structures [18]. The KAM is generally higher in people with medial knee OA and varus malalignment compared to those without [19,20]. Importantly, longitudinal studies have shown that a higher KAM is associated with the development of knee pain in older people [21] and with a 6.5-fold increase in risk of OA structural progression for a one unit increase in KAM [22]. Of major relevance is that the KAM appears to be amenable to change with non-surgical treatments such as shoes, gait modification strategies and braces by amounts that would correspond to a substantive reduction in risk of disease progression in knee OA [23-26]. Thus, the KAM is an important target outcome for treatments aimed at slowing disease progression as well as reducing symptoms. The most commonly reported indices of the KAM are the overall peak value, the two typical individual KAM peaks (early and late stance), and the area under the KAM-time curve, known as the KAM angular impulse.

As an important determinant of the KAM is frontal plane knee alignment [8], interventions which aim to reduce dynamic varus knee alignment during walking and other functional weight bearing tasks can potentially reduce the KAM. Similarly, strategies that aim to bring the frontal ground-reaction force vector closer to the knee joint centre-for example by bringing the body centre of mass closer to the knee-may also be beneficial in reducing the KAM. Although valgus knee braces are effective at improving knee alignment and reducing knee load [27,28], and thus seem a logical treatment choice, knee braces are often associated with adverse effects [29] and reduced compliance in patients with knee OA, limiting their clinical applicability [30]. In contrast, exercise is recommended by all clinical guidelines for knee OA [31,32], is associated with relatively few adverse effects [33,34] and has the potential to reduce the KAM [26].

Quadriceps strengthening has traditionally been an important component of exercise programs for knee OA. This is because quadriceps weakness is a frequent finding among people with knee OA [35-41], has been implicated in disease pathogenesis [42-44] and is associated with pain severity, physical dysfunction and functional decline [20,45]. Numerous high quality clinical trials have shown that quadriceps strength training is effective at improving pain and physical function in knee OA [46,47]. However, isolated quadriceps strengthening is ineffective at reducing pain in the subgroup of people with varus malalignment [12], nor does it reduce the KAM in those with neutral or varus malaligned knees [12,48,49]. This may be because traditional quadriceps strength training aims primarily to increase the quantity of muscle output, rather than targeting the biomechanical contributors to medial compartment knee load [50]. Thus, it is apparent that alternative exercise programs are needed to reduce knee load and alleviate symptoms for people with medial compartment OA and varus malalignment.

Neuromuscular exercise is a relatively broad class of exercise programs incorporating programs known by terms such as functional exercise, proprioceptive, agility, or perturbation training. Neuromuscular exercises are typically performed in functional weight-bearing positions emphasising quality and efficiency of movement, as well as alignment of the trunk and lower limb joints. Some also focus on elements such as responses to perturbations. Neuromuscular exercise offers promise for this patient sub-group. By focussing on improving the position of the knee in relation to the hip and ankle, specific neuromuscular exercises may enhance activation of the muscle groups most capable of generating an internal moment to counteract the external KAM during functional weight-bearing tasks. Such muscle groups include the hip adductors [51], the tensor fascia lata, lateral hamstrings, quadriceps and lateral gastrocnemius [52-55]. Furthermore, given that varus malalignment combined with ineffective dynamic muscle stability can manifest as lateral thrusting of the knee during early stance phase of walking (which is associated with increased risk of disease progression [56]), neuromuscular exercises emphasising control of lateral knee movement during weight-bearing activities may also be beneficial.

Research from other populations has demonstrated that neuromuscular exercise can affect knee functional performance, knee biomechanics and activation patterns of the surrounding knee musculature. Neuromuscular exercises are now commonly used for prevention and rehabilitation of knee injuries in young athletic individuals [57,58]. However, neuromuscular programs for younger people focus on sports specific tasks such as jumping, landing and cutting activities which are not appropriate for older individuals with knee OA. Neuromuscular training programs are thus best considered and designed as 'task-directed', that is, aimed at enhancing neuromuscular control for the specific activities of daily living important to the OA population, and their common impairments. Our neuromuscular training program is thus directed towards improving activities of daily living including walking.

There has been limited research into the benefits of neuromuscular exercise for people with knee OA with only four published studies available [26,59-61]. These are limited by mostly small sample sizes and an absence of control intervention groups. Furthermore, a relatively broad, heterogenous range of exercises have been employed, some much more vigorous than others. A single case study [59] reported improvements in pain, physical function and knee instability in a 73-year old woman with severe medial knee OA with a 6-week neuromuscular exercise program. This exercise program involved agility and perturbation techniques adapted from those prescribed for younger individuals with anterior cruciate ligament insufficiency. Using a different neuromuscular program with exercises focused on strengthening and functional activities, a pilot case series conducted in 13 people [26] showed a reduction in the KAM during a single leg sit-to-stand task following the 8 week program. Another uncontrolled feasibility study [60] showed no worsening of symptoms and few joint-specific adverse events among 38 patients with severe knee OA following a median of 13 group-delivered neuromuscular training sessions. These preliminary findings highlighted the feasibility and potential efficacy of neuromuscular exercise for reducing knee load and alleviating symptoms in people with medial knee OA and varus malalignment. However, a recent large randomised controlled trial (RCT) in 183 people with knee OA compared the addition of a neuromuscular program-particularly involving destabilizing activities designed to improve the individual's response to perturbations-to a standard strengthening exercise program. The study found that the additional program did not improve treatment effects for pain and function over and above those of the standard exercise program alone [61]. The authors surmised that there might be sub-groups of individuals who achieve an added benefit with this exercise approach. Importantly this study did not measure outcomes relevant to disease progression such as knee load. Further research is thus needed to determine the efficacy of neuromuscular exercise programs on these outcomes and for knee OA patient sub-groups.

The primary objective of this RCT is to compare the effects of a specific neuromuscular exercise program with those of traditional quadriceps strengthening exercise on the KAM, pain and physical function in an important knee OA subgroup, people with medial tibiofemoral OA and knee varus malalignment.

### Primary hypotheses

H1: The external peak KAM during walking will be reduced by a neuromuscular exercise program but not by a quadriceps strengthening program.

H2: A neuromuscular exercise program will improve self-reported physical function and reduce pain to a greater extent than a quadriceps strengthening program.

### Secondary hypotheses

H3: The KAM angular impulse during walking will be reduced by a neuromuscular exercise program but not by a quadriceps strengthening program.

H4: A neuromuscular exercise program will lead to greater improvements in muscle activation patterns, hip strength, balance, functional ability, quality-of-life and perceived change than a quadriceps strengthening program, whilst greater improvements in quadriceps strength will be found with the quadriceps strengthening program.

## Methods/Design

### Trial design

**Figure 1:**
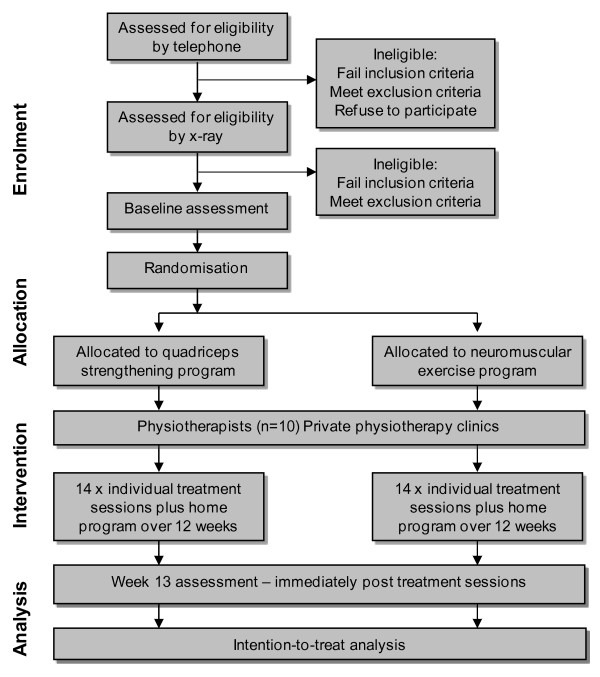
**Flow diagram of study protocol**.

Single assessor-blinded, parallel design RCT, which conforms to CONSORT guidelines for non-pharmacological studies [62] (Figure 1).

### Participants

A sample of 100 men and women aged ≥ 50 years with painful medial knee OA will be recruited from the community in metropolitan Melbourne, Australia. A number of recruitment strategies will be used including (i) advertising through local clubs, community centers, newspapers, Arthritis Australia and University websites, University staff newsletters, radio, and Facebook; (ii) placing brochures and study posters in medical and physiotherapy clinics; (iii) conducting presentations about knee OA in the local community, and (iv) using our database of people with medial knee OA and varus malalignment who were recruited from the community for prior studies and have given consent for future contact.

People will be eligible if they report average knee pain over the past week ≥ 25 on a 100 mm visual analogue scale, have predominant pain/tenderness over the medial knee region, and have radiographic evidence of medial tibiofemoral joint OA with varus knee alignment. Short limb, weight-bearing, postero-anterior radiographs will be taken with a caudal angle of 10° to achieve superimposed tibial plateau. Specific radiographic inclusion criteria are: (i) Kellgren-Lawrence grade ≥ 2 [63]; (ii) mechanical axis angle of < 181° for females or < 183° for males indicating varus alignment; (iii) medial tibiofemoral joint narrowing grade > lateral tibiofemoral joint narrowing grade [64]; and (iv) medial compartment osteophyte grade ≥ lateral compartment osteophyte grade [64]. Mechanical knee alignment will be converted from the anatomic axis measured from the knee x-ray [65] using our regression equation [66]. This method has good to excellent correlation (r = 0.65-0.88) between anatomical and mechanical axes thus avoiding the additional cost and radiation of a long limb x-ray.

Exclusion criteria will include: (i) knee surgery or intra-articular corticosteroid injection within past six months; (ii) current or past (within four weeks) oral corticosteroid use; (iii) systemic arthritic conditions; (iv) history of hip or knee joint replacement or tibial osteotomy surgery; (v) any other condition affecting lower limb function; (vi) participation in a strengthening or neuromuscular exercise program within the past six months or planning to commence exercise or other treatment for knee OA; (vii) other non-pharmacological treatment for their knee pain in the past six months including physiotherapy, acupuncture, massage therapy; or (viii) unable to ambulate without a gait aid. People who have been on glucosamine, chondroitin and/or non-steroidal anti-inflammatory drugs will not be excluded. Participants who are already wearing orthotics or insoles will be permitted to continue using them during the trial. Participants will be requested to refrain from seeking other forms of treatment during the trial.

### Procedure

Eligibility of prospective participants will be confirmed initially by telephone screening questions then on radiographic examination. Baseline and follow-up assessments will be carried out at the Department of Physiotherapy, the University of Melbourne by the same assessor who will remain blinded to exercise group allocation. Participants will visit a physiotherapist 14 times over the 12-week intervention period: twice in the first and second weeks, and weekly thereafter. Ethical approval has been obtained from the University of Melbourne Human Research Ethics Committee (HREC No. 0932813). All participants will provide written informed consent.

### Randomisation and allocation concealment

All eligible participants will be consecutively randomised into either the quadriceps strengthening program or the neuromuscular exercise program. Consecutively numbered, sealed, opaque envelopes containing exercise group allocation will be prepared by a researcher with no other involvement in the study. Exercise group allocation will be randomised within random permuted blocks of six or eight generated *a priori *using the random number function in Excel and stratified according to treating therapist so that all physiotherapists will deliver approximately equal numbers in each exercise group to control for therapist variation.

### Interventions

Ten physiotherapists in private practices at various locations throughout metropolitan Melbourne, Australia will provide the interventions. Each physiotherapist will deliver both exercise programs. The physiotherapists have an average of 13 (range 2-42) years experience since qualification and 12 (range 2-30) years of post-graduate clinical musculoskeletal experience. Three (30%) have postgraduate Masters qualifications in sports or manipulative/musculoskeletal therapy. The physiotherapists will attend a three hour training session covering delivery of both exercise programs and receive a detailed treatment manual describing each exercise intervention. After initiation of the trial, telephone meetings will be held to discuss issues experienced in the clinic and solutions will be suggested. This procedure will reinforce similar treatment administration among therapists. Physiotherapists will be supplied with weights and elastic bands to provide to the study participants.

One knee will be the focus of the treatment and evaluated. Focussing on one knee only is to minimise the burden of exercise and laboratory testing time. If participants have bilateral symptoms, the most symptomatic eligible knee or the right knee, in the case of equally symptomatic knees, will be nominated. Some of the neuromuscular exercises are performed weight-bearing on both legs, while other exercises are performed weight-bearing on one leg. In the latter, the weight-bearing leg is the affected leg. In contrast, the quadriceps strengthening exercises are performed in non-weight-bearing positions with the affected leg only.

Each supervised exercise session in both programs will last 30-40 minutes. Participants in both groups will be asked to perform their prescribed exercises at home four times per week in addition to performing them at the scheduled supervised physiotherapy visits. A brief re-assessment will be performed by the physiotherapist at each physiotherapy session in order to ascertain any adverse effects occurring during the preceding week and to check quality and form of exercise performance. The findings from this assessment will help guide physiotherapists' decisions regarding progression of the exercises.

#### Neuromuscular exercises

As alluded to in the introduction, the neuromuscular exercises aim to improve the position of the trunk and lower limb joints relative to one another, as well as quality of movement performance, while dynamically and functionally strengthening the lower limb muscles. As it would be difficult to focus participants' attention on preferentially activating muscle groups that can counteract varus malalignment (eg. the hip adductors [51], the tensor fascia lata, lateral hamstrings, quadriceps and lateral gastrocnemius [52-55]) in isolation, a more pragmatic approach to training will be adopted whereby participants will be instructed to improve control of knee and hip muscles by practicing more neutral knee positioning during a series of specified exercises/tasks. The participants will be instructed to aim at positioning their knee over the foot, and to avoid a medial or lateral position of the knee in relation to the foot. It is acknowledged however, that a knee over foot position may not be achievable for people with varus malalignment. Lateral thrusting of the knee is to be avoided and controlled at all times as much as possible. Specific exercises have been selected on the basis that they involve movement of the knee in synergy with all joints in the lower extremity, are weight-bearing (closed-chain) and are functionally relevant. Participants will be instructed to also focus on maintaining neutral pelvic alignment during performance of the exercises. It was felt important to include at least one exercise that would challenge postural stability and thereby encourage activity of anti-gravity postural muscles. The selected exercises were developed from a range of sources [26,60,67].

Progression will be provided by varying the repetitions, direction, and velocity of the movements; increasing the load; and/or changing the support surface. Each of the six exercises in the program and their levels and frequencies are described in detail in Additional File 1. Participants in the neuromuscular exercise group will be made aware of the following points:

• Quality of performance is critical and the participant must attempt to position their knee over the foot throughout the movements.

• Knee flexion should not exceed 30° during the exercises (except when performing the chair stand exercise). This was to minimise the risk of increasing knee pain and is relevant to the range of knee flexion during walking, our primary outcome.

• Although some discomfort is expected, the exercises should be performed within tolerable levels of pain. Pain should subside to usual levels by the next day with no increase in swelling following the exercise session. Participants are assisted in determining whether pain levels during and for a short time after the exercises are acceptable by using a pain monitoring scale where zero is "no pain" and ten is "pain as bad as it could be". Pain up to two on the scale is considered 'safe', pain up to a level of five is considered 'acceptable' as long as temporary, and pain above five is considered 'high risk' [60].

• Safety should be ensured by using hand support or having hand support within easy reach. This is used for balance support and for maintaining quality of performance throughout the movements.

• Participants should be challenged by the exercises. During all exercises, the level of effort experienced should be self-rated as at least 5 out of 10 on a modified Borg Rating of Perceived Exertion (RPE) CR-10 scale [68].

If the physiotherapist considers that a specific exercise is aggravating the participant's pain, then the therapist will reduce the resistance, dosage and/or level of the exercise until the pain flare settles. Progression of exercises is an essential component of the program and will be determined by the physiotherapists based on their assessment of the quality of the exercise performance, on the RPE score for each exercise and on the participant-reported pain response. All participants should be progressed during the treatment phase of the study, although it is expected that not all will reach the final progression stage on all exercises.

#### Quadriceps strengthening

The aim of the program is to improve the strength of the quadriceps and is based on the program in our previous RCT [12]. The participants in the quadriceps strengthening group will complete five specific non-weight-bearing exercises with the affected (most symptomatic or right) leg:

1. Quads over a roll (inner range knee extension)-using resistance of ankle weights.

2. Knee extension in sitting-start sitting with knee at 90° flexion, fully extend using resistance of ankle weights.

3. Knee extension with hold at 30° knee flexion-start sitting with knee at 90° flexion, extend to 30° using resistance of ankle weights.

4. Straight leg raise-start supine, raise leg to 30° hip flexion using resistance of ankle weights.

5. Elastic band exercise-start sitting with knee at 90° flexion, extend to 60° against resistance of elastic band.

Ten repetitions will be performed in each set of quadriceps exercises. Two sets will be performed at the start of the program, progressing to three sets as quickly as possible. The starting weight should be the participant's 10-repetition maximum weight if possible. However, the starting weight can also be determined by asking the participant their level of effort which should be 5-8 out of ten (hard to very hard) on the modified Borg RPE CR-10 scale for strength training [68]. Each repetition will be performed slowly and in a controlled manner. The end position is held for five seconds initially and progressed to ten seconds. Breath holding during the isometric component of the exercises can increase blood pressure; therefore participants will be instructed to continue breathing throughout each phase (concentric, isometric and eccentric) of the exercises. Exercises should be carried out within tolerable levels of pain. The same pain monitoring scale as described above will be used and pain should subside to usual levels by the next day with no increase in swelling following the exercise session. If joint swelling or 'unacceptable' pain occurs, the resistance, frequency and/or number of repetitions will be reduced. Progression is again an important part of the program and participants will aim to increase their ankle weight or elastic band resistance at regular intervals during the program as guided by their physiotherapist. During all exercises, the level of effort experienced should be self-rated as at least five out of ten (hard) on a RPE CR-10 scale for strength training [68].

### Treatment integrity

Study physiotherapists will keep standardised treatment notes. Selected treatment sessions will be attended by a researcher to document adherence to the protocol. Participants will be questioned at the end of their treatment about their physiotherapy treatment experience.

### Outcome measures (Table 1)

#### External knee adduction moment

Participants will undergo a 3D gait analysis to assess dynamic loading of the knee during walking at a self-selected speed. Movement will be recorded using a 12-camera motion analysis system (Vicon MX, Oxford, UK) and force plates (AMTI, MA, USA) as participants walk barefeet along a 10 m level walkway with speed monitored by two photoelectric beams. Five successful trials (complete foot strike from one foot on a force plate) will be obtained for each leg. The motion of reflective markers (sample rate 120 Hz) and the ground reaction force (sample rate 1200 Hz) will be used to calculate the external KAM using inverse dynamics via the University of Western Australia (UWA) model, programmed in Vicon Body Builder [69]. Test-retest reliability (coefficient of multiple determination, *r*^2^) of knee adduction/abduction moment curves averaged over six trials using UWA model was reported as at least 0.75 [69]. The primary variable of interest is the overall peak KAM normalised for body weight times height (Nm/BW*HT%) [70] and averaged over the five trials. KAM angular impulse (the positive area under the KAM-time curve) will be calculated as a secondary outcome variable.

**Table 1:** Summary of measures to be collected.

Primary outcome measures	Data collection instrument
External peak knee adduction moment (KAM)	3-dimensional gait analysis system and University of Western Australia (UWA) functional model
Average overall pain in past week	100 mm visual analogue scale
Physical function in past 48 hours	WOMAC Osteoarthritis Index 3.1 Likert version physical function subscale

**Secondary outcome measures**	

KAM angular impulse	3-dimensional gait analysis system and (UWA) functional model
Pain and stiffness	WOMAC Osteoarthritis Index 3.1 Likert version
Participant global rating of change overall and for pain and function	7-point ordinal scale
Muscle activation and co-contraction patterns	Surface electromyography during walking
Hip and knee muscle strength	Isometric knee flexors and extensors (isokinetic dynamometer), hip abductors and rotators (instrumented manual muscle tester), and hip extensors (force transducer).
Physical performance	Single limb standing time (seconds)
	Step test
	Four square test
	Timed stair climb (ascent and descent)
	30 second sit-to-stand test
Health-related quality of life	Assessment of Quality of Life Instrument version 2 (AQoL II)

**Other measures**	

Mechanical knee alignment	X-ray (baseline)
Disease severity	X-ray (baseline)
Physical activity levels	Physical Activity Scale for the Elderly (PASE)
Adverse events	Participant log-book (follow-up)
Adherence/Treatment session attendance	Participant log-book (follow-up)
	Therapist treatment records (follow-up)

All measures recorded at baseline and follow-up (week 13) unless otherwise stated.

#### Self-reported pain and physical function

The primary pain outcome is average overall knee pain during the past week. This, together with pain on walking during the past week, will be assessed using 100 mm visual analogue scales with terminal descriptors of "no pain" and "worst pain possible". Such measurement has demonstrated reliability in OA [71]. Pain will also be assessed, along with stiffness and physical function, using the disease-specific Western Ontario McMaster Universities (WOMAC) Osteoarthritis Index [72]. The physical function subscale, which comprises 17 questions, will be used as a primary outcome measure of self-reported physical function.

At the follow-up assessment, participants will rate their perceived a) overall change, as well as change in b) pain and in c) physical function with the exercise program (compared to baseline) on a seven-point ordinal scale (1-much worse to 7-much better). Scales of this kind are frequently used as an external criterion for comparison with changes in scores of other outcomes [73]. Measuring participant-perceived change using a rating of change scale has been shown to be a clinically relevant and stable concept for interpreting truly meaningful improvements from the individual perspective [74].

#### Muscle co-contraction and activation patterns

Muscle activity recordings will be made during the walking trials using surface electromyography (EMG) from lateral muscles (biceps femoris, lateral gastrocnemius, tensor fascia lata), medial muscles (medial hamstrings, medial gastrocnemius) and quadriceps (vastus medialis, vastus lateralis, rectus femoris). EMG signals will be band-pass filtered between 20 Hz-500 Hz and sampled at 1200 Hz synchronously with the Vicon camera data via a telemetered 8-channel Noraxon Telemyo 9000 system (Noraxon, AZ, USA). EMG recordings during maximal isometric knee flexion, knee extension, hip abduction and plantar flexion will be used for normalisation of EMG data. Total activation, relative activation of individual muscles and abducting/adducting muscle groups, and co-contraction, will be assessed.

#### Muscle strength

Maximum, normalised, isometric strength (Nm/kg) will be recorded for key hip and knee muscle groups. Quadriceps and hamstring strength will be measured at 60° knee flexion in sitting using an isokinetic dynamometer (KinCom 125-AH, Chattanooga Corp, TN, USA). Participants will perform 3 maximal contractions for a period of five seconds each with the best of the 3 trials being used for the analysis. Isometric hip abductor and hip internal and external rotation muscle strength will be measured using a hand held dynamometer (Lafayette Manual Muscle Test System 01163, Lafayette, IN). For hip abduction, the participant will lie supine with the hip in a neutral position. For internal and external rotation, the participant will sit with the hip and knee at 90° flexion. Isometric hip extensor strength will be measured using a ceiling mounted Shimpo FGC-50 force transducer (Nidec-Shimpo, Kyoto, Japan) and digital inclinometer (SmartTool, MD Building Products, OK, USA), with the participant in supine and their hip in 20° flexion. For the hip strength measurements, the mean of two maximal trials will be used in the analysis [75]. During all strength measurements strong verbal encouragement will be given and this is standardised between participants.

#### Physical performance measures

Balance tests-1) Single limb standing balance will be timed (seconds) up to 30 seconds [76]. The best attempt from two trials will be recorded. 2) The number of steps by the non-study leg onto a 15 cm high step and back to the floor in 15 sec will be recorded with the participant carrying out the task as quickly as possible (Step test) [77,78]. 3) The Four-square step test will also be performed, where two sticks are used to make a cross shape on the floor and the time taken to step from quadrant 1 to 2, then to 3, 4, 1, 4, 3, 2 and 1 again as quickly as possible is recorded [79].

Timed stair climb-The time (seconds) to walk up and down six 17.5 cm high steps as quickly as possible, using a hand rail if they prefer, will be recorded [80].

Thirty second sit-to-stand test-The number of sit-stand-sits in 30 seconds achieved with the participant carrying out the task as quickly as possible will be recorded [81]. The task will be performed on a standard height chair without use of upper limbs.

#### Health-related quality of life

Health-related quality of life will be measured using the Assessment of Quality of Life instrument version two (AQoL II). The AQoL II has 20 questions that cover six dimensions of health-related quality of life including independent living, social relationships, physical senses, coping, pain and psychological wellbeing. The AQoL has strong psychometric properties and is more responsive than other widely-used scales [82,83]. It produces a single utility index that ranges from -0.04 (worst possible health-related quality of life) to 1.00 (full health-related quality of life). A clinically important difference in health-related quality of life can be defined as a change of 0.04 AQoL units [84].

#### Other measures

Disease severity will be gauged from the baseline knee x-ray and classified using the Kellgren-Lawrence grading system [63]. Baseline demographic information including social factors, medication use, co-morbidities and other treatments will be recorded as well as measures taken of height and weight. Co-interventions, adherence and adverse effects will be determined from the participants' log books and the physiotherapists' treatment notes.

Habitual physical activity will be measured using the Physical Activity Scale for the Elderly (PASE), a self-report questionnaire that has been shown to be reliable, valid and sensitive to change in people with knee OA [85,86]. It records both the level and type of recreational and occupational physical activity undertaken by participants over the previous week. The PASE was developed and validated in samples of older adults (age 55+ years) [87].

### Sample size

Our three primary endpoints are the overall peak KAM during the stance phase of walking, VAS overall knee pain, and WOMAC physical function scores. The minimum clinically important difference to be detected for a change in KAM is unknown. However, a reduction in KAM of 7.5% may be associated with a significant decrease in the risk of disease progression, based on the results of Miyazaki et al [22], who found an increased progression risk of over 6 times associated with an approximately 20% greater KAM. This magnitude of reduction appears to be achievable with exercise as a pilot study showed a 14% reduction with similar neuromuscular exercise in knee OA [26]. The minimum clinically important difference to be detected in OA trials is a change in pain of 18 mm on VAS [88] and a change of six physical function WOMAC units (out of 58) [89]. Based on our previous data, we assume a common between-participant standard deviation of change in KAM of 0.4 Nm/BW*HT%, 30 mm for pain, and 12 units for WOMAC physical function. These statistics indicate a smaller standardized effect size of interest (Cohen's *d*) of 0.5 for physical function than the *d *= 0.6 for pain and *d *= 0.75 for KAM. Given this, the required sample for a two-tailed comparison of the two exercise groups using analysis of covariance with baseline values as covariates, when *d *= 0.5, power is 0.8 and type I error is .05 is 41 participants per group. To allow for a 15% dropout rate a total of 100 participants will be recruited.

### Data and statistical analysis

Main comparative analyses between groups will be performed in a blinded fashion using an intention-to-treat approach with p-values of less than 0.05 considered significant. To account for missing data, multiple imputation of missing follow-up measures, assuming missing data are missing at random and follow a multivariate normal distribution [90], will be performed as a sensitivity analysis. For continuous outcome measures, differences in mean change (baseline minus follow-up) will be compared between groups using analysis of covariance adjusted for baseline values of the outcome. Walking speed will also be included as a covariate for the KAM parameters if follow-up walking speed differs. Model diagnostic checks will utilise residual plots. Results will be presented as estimated differences with 95% confidence intervals [91]. We will also perform a per protocol analysis as appropriate. Effect sizes will be calculated for all measures with an effect size of 0.2 considered small, 0.5 medium and 0.8 large [92]. Participant perceived overall change and change in pain and in physical function will be compared between groups using log binomial regression. Results will be presented as relative risks with 95% confidence intervals.

### Timeline

Ethics approval was obtained in April 2010 from the Human Research Ethics Committee of the University of Melbourne. Recruitment and training of the physiotherapists was undertaken in May 2010 and recruitment of participants has commenced. All participants are expected to have completed the study by end 2012.

## Discussion

The need to develop efficacious treatment approaches for knee OA that are capable of not only ameliorating symptoms but also slowing disease progression is an important research and clinical objective [93]. Our study is based on the premise that while the static structural malalignment itself cannot be altered in individuals with medial knee OA, except via surgical procedures, other factors contributing to higher knee load are potentially modifiable with exercise interventions. If the loading forces can be reduced within the medial tibiofemoral compartment during weight bearing, structural degeneration may be slowed in addition to achieving symptom relief.

Our study is the first RCT to investigate the effect of neuromuscular exercise on knee load, pain and function in people with medial knee OA and varus malalignment. Strengths of the study design are the pragmatic nature of treatment delivery which occurs in community physiotherapy clinics by several practicing physiotherapists, and the reproducibility of both exercise programs. These features will improve the generalisabilty of the findings. Importantly however, both programs are individualised with regard to the level of exercise intensity and both incorporate progression. In addition, the study is adequately powered for all three primary outcome measures and our recruitment strategy will result in a well characterised, homogenous sample.

This trial evaluates an innovative neuromuscular exercise program that aims to reduce medial knee load and pain and improve function in people with medial compartment OA and varus malalignment. The findings may lead to a more effective exercise treatment option than currently exists for this important subgroup of people with knee OA.

## Competing interests

The authors declare that they have no competing interests.

## Authors' contributions

KLB, MH and RH conceived the project and KLB is leading the co-ordination of the trial. KLB, RH, TW, PH, MH, ER, AF and EA assisted with protocol design and procured the project funding. TW and BM designed the biomechanical and physical impairment measures. KLB, RH, PH, MH, EA and ER designed the neuromuscular exercise program and KLB and RH trained the therapists. AF performed the sample size calculations and designed the statistical analyses. KLB and TE wrote the first draft of the manuscript. TE wrote the protocol manual and the final drafts of this manuscript. BM is the blinded assessor on the project while MK recruits and screens the participants and manages the project. All authors participated in the trial design, provided feedback on drafts of this paper and read and approved the final manuscript.

## Pre-publication history

The pre-publication history for this paper can be accessed here:

http://www.biomedcentral.com/1471-2474/12/276/prepub

## Supplementary Material

Additional file 1 Neuromuscular exercises. Click here for file
