# Supplementary material for: Comparison of neuromuscular and quadriceps strengthening exercise in the treatment of varus malaligned knees with medial knee osteoarthritis: a randomised controlled trial protocol
Source: BMC Musculoskelet Disord. 2011 Dec 5;12:276. doi: 10.1186/1471-2474-12-276 (PMC3247187; doi:10.1186/1471-2474-12-276)
Supplement: Additional file 1 — Neuromuscular exercises. [file 1471-2474-12-276-S1.doc]

### Additional file

### Neuromuscular exercises.

The neuromuscular exercise program with levels and repetitions. Progression through the levels is an important component of the program. As a guide, participants should be progressed after 3 weeks at a level provided they have good form and the level is not aggravating their pain.

|  |  | **Level** | **Repetitions** |
| --- | --- | --- | --- |
| **Exercise 1. Forwards/ backwards** | | | |
| Level 1:  Slide the non-arthritis leg backwards and forwards while bending and straightening the arthritis leg. Arthritis leg is nearly straight when feet are together and bent when feet are apart. | Level 1 | 1. Sliding forwards/backwards (aim at positioning knee over foot) | 3 sets of 10 with break of 30-60 seconds between sets |
|  |  | 2. Sliding with elastic band  (Choose elastic band colour appropriate for the participant’s ability to achieve a knee over foot position. Attach the band to a table leg on the arthritis leg side) | 3 sets of 10 with break of 30-60 seconds between sets |
|  |  | 3. Stepping forwards/backwards (aim at positioning knee over foot) | 3 sets of 10 with break of 30-60 seconds between sets |
|  |  | 4. Stepping with elastic band  (Choose elastic band colour appropriate for the participant’s ability to achieve a knee over foot position. Attach the band to a table leg on the side of the arthritis leg) | 3 sets of 10 with break of 30-60 seconds between sets |
| **Exercise 2. Sideways exercise** | | | |
| Level 1:  Slide the non-arthritis leg sideways while bending and straightening the arthritis leg.  Arthritis leg is nearly straight when feet are together and bent when feet are apart. | Level 1 | 1. Sliding sideways, out and in (aim at positioning knee over foot) | 3 sets of 10 with break of 30-60 seconds between sets |
|  |  | 2. Sliding with elastic band  (Choose elastic band colour appropriate for the participant’s ability to achieve a knee over foot position. Attach the band to a table leg on the arthritis leg side) | 3 sets of 10 with break of 30-60 seconds between sets |
|  |  | 3. Sliding with elastic band and soft surface. (As above while standing on a pillow or cushion) | 3 sets of 10 with break of 30-60 seconds between sets |
|  |  | 4. Sliding with elastic band, soft surface and eyes closed | 3 sets of 10 with break of 30-60 seconds between sets |
| **Exercise 3. Hip muscle strengthening** | | | |
| Level 1, Wall push:  Standing sideways to a wall with non-arthritis leg closest to wall so that hip, thigh and knee are all slightly bent and touching the wall. Slightly bend arthritis knee (15-20°). Push leg into the wall and hold.  Activity (Level 3, Crab walk):  With elastic band around ankles, step sideways in both directions maintain slight knee bend throughout. | Level 1    Level 3 | 1. Wall push | 20 second holds with short break between efforts. Two sets of 5 with break of 30-60 seconds between sets. |
|  |  | 2. Wall push with knee bending [Slowly bend and straighten arthritis knee while maintaining the push.] | Short break between efforts. Two sets of 5 with break of 30-60 seconds between sets. |
|  |  | 3. Crab walking with red elastic band | Total of 30 steps in each direction. |
|  |  | 4. Crab walking with black elastic band | Total of 30 steps in each direction. |
| **Exercise 4. Knee muscle strengthening** | | | |
| Level 1, Wall squats:  Stand with back to a wall, feet 10cm apart and one foot length away from the wall. Slide slowly down the wall to about 30° bend, then slide up again.  Level 3, Chair stands:  Start sitting on a standard height (eg. kitchen) chair. Reach hands out in front. Stand up and sit down slowly. | Level 1    Level 3 | 1. Wall squats (aim at positioning knee over foot) | 3 sets of 10 with break of 30-60 seconds between sets |
|  |  | 2. Wall squats with more weight on arthritis leg (shift weight more over arthritis leg) | 3 sets of 10 with break of 30-60 seconds between sets |
|  |  | 3. Chair stands (aim at positioning knee over foot) | 3 sets of 10 with break of 30-60 seconds between sets |
|  |  | 4. Chair stands with more weight on arthritis leg | 3 sets of 10 with break of 30-60 seconds between sets |
| **Exercise 5. Steps** | | | |
| Level 1, Step-ups:  Place arthritis leg onto a step. Slowly step up onto the step. Touch non-arthritis side foot to the step then step back down slowly to the start position.  Level 3, Touch downs:  Start standing on the step. Touch non-arthritis side foot to the floor in front and then behind the step. | Level 1    Level 3 | 1. Step-ups (aim at positioning knee over foot) | 3 sets of 10 with break of 30-60 seconds between sets |
|  |  | 2. Step-ups with 2kg weight or higher step (aim at positioning knee over foot) | 3 sets of 10 with break of 30-60 seconds between sets |
|  |  | 3. Touch downs (aim at positioning knee over foot) | 3 sets of 10 with break of 30-60 seconds between sets |
|  |  | 4. Touch downs with 2kg weight or higher step (aim at positioning knee over foot) | 3 sets of 10 with break of 30-60 seconds between sets |
| **Exercise 6. Balance** | | | |
| Level 1:  Lift non-arthritis side foot off the floor and balance. | Level 2 | 1. Standing on one leg (aim at positioning knee over foot) | 2 minutes practice |
|  |  | 2. Step forward to standing on one leg (aim at positioning knee over foot) | 2 minutes practice |
|  |  | 3. Step to standing on one leg and add arm movements (aim at positioning knee over foot) | 2 minutes practice |
|  |  | 4. Step to standing on one leg on soft surface (aim at positioning knee over foot) | 2 minutes practice |
